# Herbal medicine for acute management and rehabilitation of traumatic brain injury: A protocol for a systematic review

**DOI:** 10.1097/MD.0000000000014145

**Published:** 2019-01-18

**Authors:** Boram Lee, Jungtae Leem, Hyunho Kim, Hee-Geun Jo, Sang-Hoon Yoon, Aesook Shin, Jae-Uk Sul, Ye-Yong Choi, Younghee Yun, Chan-Young Kwon

**Affiliations:** a Department of Korean Medicine, Kyung Hee University Korean Medicine Hospital at Gangdong, Seoul; b Chung-Yeon Medical Institute, Gwangju; c Dongshin Korean Medicine Hospital, Seoul; d Chung-Yeon Korean Medicine Hospital, Gwangju; e Research and Development Institute, CY Pharma Co.; f Department of Clinical Korean Medicine, Graduate School, Kyung Hee University, Seoul, Republic of Korea.

**Keywords:** herbal medicine, protocol, systematic review, traumatic brain injury

## Abstract

**Background:**

This systematic review protocol describes the methods that will be used to evaluate the efficacy and safety of herbal medicine in treating traumatic brain injury.

**Methods and analysis:**

The following electronic databases will be searched up to December 2018 without language or publication status restrictions: Medline, EMBASE, the Cochrane Central Register of Controlled Trials, Allied and Complementary Medicine Database, and Cumulative Index to Nursing and Allied Health Literature. We will also search Korean, Chinese, and Japanese databases. Any randomized controlled trials related to herbal medicine for traumatic brain injury will be included. The functional outcome, consciousness state, morbidity, and mortality will be assessed as primary outcomes. The quality of life, adverse events, and total effective rate will be evaluated as secondary outcomes. Two researchers will independently perform the study selection, data extraction, assessment of study quality, and evaluation of the quality of evidence for the main findings. Data synthesis and analysis will be performed using RevMan version 5.3. The results will be expressed as a risk ratio for the binary outcome and as the mean difference or standardized mean difference for a continuous outcome. We will synthesize the data by either fixed effects or random effects model according to a heterogeneity test or the number of studies included in the meta-analysis. The methodological quality of the included studies will be evaluated using the Cochrane Collaboration's risk of bias tool. The quality of evidence for each main outcome will be evaluated using the Grading of Recommendations Assessment, Development, and Evaluation approach.

**Ethics and dissemination:**

Ethical approval is not required because individual patient data are not included. The findings of this systematic review will be disseminated through a peer-reviewed publication or conference presentations.

**PROSPERO registration number:**

CRD42018116559.

## Abstract 2

Supplemental Digital Content is available in the text

## 1 Introduction

Traumatic brain injury (TBI), defined as *“*an alteration in brain function, or other evidence of brain pathology, caused by an external force*”*,^[1]^ is considered a leading cause of death and disability worldwide. Although global epidemiology has not yet been well characterized, a recent systematic review of 82 population-based studies reported that TBI occurred in approximately 300 cases per 100,000 people every year.^[2]^ According to the Centers for Disease Control and Prevention (CDC) data^[3]^ and epidemiological studies in Europe,^[4,5]^ the most common causes of TBI are falls and road traffic accidents; the former is common in children and in the elderly, and the latter is common in young adults.^[3]^

The severity of TBI varies according to the site affected and severity of the injury, but it can be classified as mild, moderate, or severe by measures such as the Glasgow Coma Scale (GCS), which assesses the state of consciousness, duration of the loss of consciousness, and post-traumatic amnesia.^[6]^ TBI reduces the quality of life (QoL) of affected individuals and is related to several negative long-term consequences such as neurological complications, physical disabilities, neurodegenerative diseases, including dementia and Parkinson diseases, and epilepsy.^[7–9]^ More importantly, the recent CDC report on TBI announced that one-fifth of patients receiving inpatient rehabilitation with moderate-to-severe TBI die within 5 years, and almost half of them experience a change in their cognitive function between 1 and 5 years post-injury.^[10]^ In particular, many people have a post-concussion syndrome (PCS), a complex of symptoms such as headache, dizziness, cognitive impairment, and neuropsychiatric symptoms, as sequelae after mild TBI.^[11]^

The acute management of TBI has improved dramatically over the past decades due to the development of surgical techniques; however, there remains a lack of optimal neurorestoration and neurorehabilitation management in TBI survivors with disabilities.^[12]^ In addition, pharmacological treatment did not show significant superiority to placebo in treating TBI, although this may also be evidence that the placebo effect is important for TBI recovery.^[13]^ Therefore, the need for effective and safe new therapeutic approaches in the management of TBI should be emphasized.

In this regard, some complementary and integrative medicine (CIM) modalities, such as acupuncture and herbal medicine (HM), are attracting attention as new alternatives in TBI treatment. Although a Cochrane review on acupuncture for TBI in 2013 could not draw firm conclusions because of the poor quality of the included studies,^[14]^ subsequent studies suggested some benefits of acupuncture for TBI management, for example, by reducing the number of TBI-related emergency room visits and hospitalizations^[15]^ and the incidence of strokes.^[16]^ Moreover, in a recent systematic review of 15 randomized controlled trials (RCTs) evaluating the efficacy of HM on hemorrhage-related hydrocephalus, including TBI-induced hydrocephalus, HM showed potentially beneficial effects in improving ventriculomegaly and clinical signs and symptoms.^[17]^ In addition, clinical evidence of some HMs or HM products, such as Yokukansan^[18]^ and NeuroAid,^[19]^ on TBI have been reported. HM has the potential to have beneficial effects on TBI through multidimensional pathways, such as reducing brain water content, improving blood–brain barrier permeability, and reducing tumor necrosis factor-α/nitric oxide expression.^[20]^

Development of new alternatives, including HM, may be helpful in establishing an optimal TBI management strategy, and efforts to comprehensively collect and critically evaluate relevant, current research are needed. However, because the efficacy and safety of HM for TBI have not yet been systematically examined, we will review the potential roles of HM for TBI based on previously published literature, focusing especially on PCS, which is a chronic sequela of TBI.

## 2 Methods

### 2.1 Study registration

The protocol for this systematic review has been registered in the International Prospective Register of Systematic Reviews, PROSPERO (registration number, CRD42018116559) on December 6, 2018. We will conduct a systematic review according to this protocol, but if protocol amendments occur, the dates, changes, and rationales for each amendment will be tracked in PROSPERO. This protocol is reported in accordance with the Preferred Reporting Items for Systematic Review and Meta-Analysis Protocols 2015 statement^[21]^ and the Cochrane Handbook for Systematic Reviews of Interventions.^[22]^

### 2.2 Data sources and search strategy

The following databases will be searched comprehensively and systematically from their inception dates to December 2018: 5 English-language databases (Medline via PubMed, EMBASE via Elsevier, the Cochrane Central Register of Controlled Trials, the Allied and Complementary Medicine Database via EBSCO, and the Cumulative Index to Nursing and Allied Health Literature via EBSCO), 5 Korean-language databases (Oriental Medicine Advanced Searching Integrated System, Koreanstudies Information Service System, Research Information Service System, Korean Medical Database, and Korea Citation Index), 3 Chinese-language databases (China National Knowledge Infrastructure, Wanfang Data, and VIP), and 1 Japanese database (CiNii). In addition, we will search the reference lists of the relevant articles and perform a manual search on Google Scholar to identify further studies. We will include the literature published in journals and also “gray literature” such as degree theses and conference proceedings. There will be no restriction on language, publication date, or publication status.

The search terms will be composed of the disease term part and the intervention term part. The search strategies for the Medline are shown in Table 1 and will be modified and used similarly in the other databases.

**Table 1:**
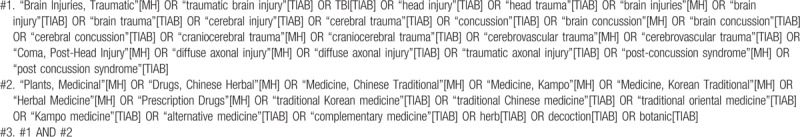
Search strategies for the Medline.

### 2.3 Inclusion criteria

#### 2.3.1 Types of studies

We will include RCTs only. We will exclude studies using inappropriate random sequence generation methods such as alternate allocation or allocation by birthdate. If the expression “randomization” (randomization) is only mentioned without the randomization methods, it will be excluded in this review. We will include both parallel and crossover studies. In crossover studies, only first-phase data will be used to calculate the effect size and conduct the meta-analysis. Other designs, such as in vivo, in vitro, case reports, and retrospective studies, will be excluded.

#### 2.3.2 Types of participants

We will include studies on patients having TBI. There will be no restriction on the severity of TBI, sex, age, or race of the participants. Studies will be excluded if the participants have drug allergies or other serious medical conditions such as cancer, liver disease, or kidney disease.

#### 2.3.3 Types of interventions

We will include only those studies using HM as experimental interventions. We will allow any formulation of HM (eg, decoction, tablets, capsules, pills, powders, and extracts); however, we will only include studies in which HM is administered orally. Except for patent medicines, studies that do not list the composition of HM used will be excluded. We will exclude studies comparing different types of HM because these studies cannot yield and demonstrate the net effect of HM. As control interventions, we will include placebo, no treatment, and conventional medical treatments, for example, acute management and rehabilitation, (which may be baseline treatments) for TBI. In our study, acute management involves the stabilization of patients immediately after the injury; the time frame may be from the onset of injury to 1 month following injury. Rehabilitation involves the treatment of long-term impairments and the return of patients to the community; the time frame may be from 1 month to 2 years following injury.^[14]^ Studies involving HM combined with other therapies as experimental interventions will be included if the other therapies are used equally in both the experimental and control groups. No other restrictions will be placed on control interventions.

#### 2.3.4 Types of outcome measures

The primary outcome measures are as follows:

(1) Functional outcome measured by validated scales, such as the Barthel Index,^[23]^ Functional Independence Measurement,^[24]^ Fugl-Meyer Assessment,^[25]^ or Glasgow Outcome Scale^[26]^
(2) Consciousness state measured by validated scales such as GCS^[27]^
(3) Morbidity
(4) Mortality

The secondary outcome measures are as follows:

(1) QoL measured by validated assessment tools such as the 36-Item Short Form Health Survey^[28]^
(2) Adverse events as measured by the Treatment Emergent Symptom Scale^[29]^ or the incidence
(3) Total effective rate

The total effective rate is a non-validated outcome measure that is processed secondarily according to certain evaluation criteria such as clinical symptom improvement or improvement rates of other quantified outcomes. In the assessment of the total effective rate, participants are generally classified as “cured” (cured), “markedly improved” (markedly improved), “improved” (improved), or “non-responder” (non-responder) after treatment. The total effective rate is calculated consistently using the following formula:

Total effective rate *=* *N*1* + N*2* + N*3/*N,* where *N*1, *N*2, *N*3, and *N* are the number of patients who are cured, markedly improved, improved, and who comprise the sample size, respectively.

### 2.4 Study selection

**Figure 1:**
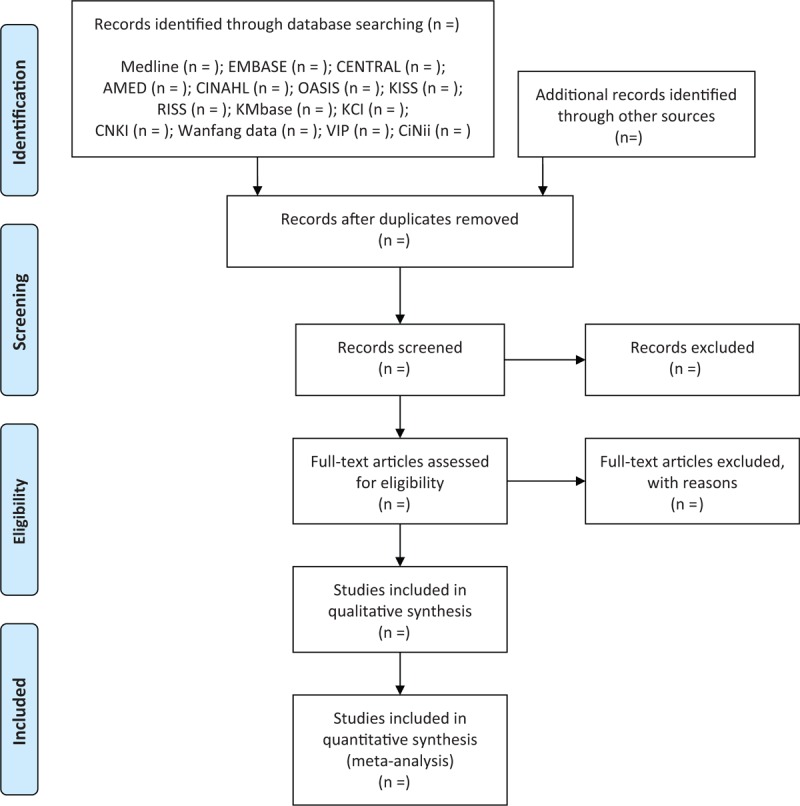
A PRISMA flow diagram of the literature screening and selection processes. AMED = Allied and Complementary Medicine Database, CENTRAL = Cochrane Central Register of Controlled Trials, CINAHL = Cumulative Index to Nursing and Allied Health Literature, CNKI = China National Knowledge Infrastructure, KCI = Korea Citation Index, KISS = Koreanstudies Information Service System, KMbase = Korean Medical Database, OASIS = Oriental Medicine Advanced Searching Integrated System, RISS = Research Information Service System.

Two researchers (B L and C-Y K) will independently conduct the study selection according to the above inclusion criteria. After removing duplicates, we will examine the titles and abstracts of the searched studies for relevance and then evaluate the full texts of the remaining studies for final inclusion. Any disagreement between 2 researchers will be resolved through discussion with other researchers. Quotations from included articles will be made available to researchers using EndNote X8 (Clarivate Analytics, Philadelphia), a reference management software program. We will report the study selection process according to the Preferred Reporting Items for Systematic Reviews and Meta-Analyses statement (Fig. 1).^[30]^

### 2.5 Data extraction

Using a standardized data collection form in Excel 2007 (Microsoft, Redmond, WA), 2 researchers (B L and C-Y K) will independently perform and double-check the data extraction. Discrepancies will be resolved through discussion with other researchers.

We will extract the following items: the first author's name; publication year; country; approval of institutional review board; informed consent; sample size and number of dropouts; diagnostic criteria such as confirmation by brain computed tomography, magnetic resonance imaging, or diffuse tensor imaging^[31]^ and international statistical classification of diseases and related health problems or diagnostic and statistical manual of mental disorders for the diagnosis of PCS; details about the participants, intervention, and comparisons; duration of the intervention and follow-up; outcome measures; outcomes; and adverse events. In particular, regarding the data on HM, the name, source, dosage form, and dosage of each medical substance, as well as the principles, rationale, and interpretation of the intervention form will be extracted by consulting the Consolidated Standards of Reporting Trials Extension for Chinese Herbal Medicine Formulas 2017.^[32]^

We will share the extracted data among researchers using Dropbox (Dropbox, Inc, CAalifornia) folders. We will contact the corresponding authors of the included studies via E-mail or telephone to request additional information if the data are insufficient or ambiguous.

### 2.6 Quality assessment

Two researchers (B L and C-Y K) will independently evaluate the methodological quality of the included studies and the quality of evidence for main findings. We will solve discrepancies between 2 researchers through discussion with other researchers.

The methodological quality of the included studies will be assessed using the Cochrane Collaboration's risk of bias tool.^[22]^ We will assess random sequence generation, allocation concealment, blinding of participants, personnel, and outcome assessors, completeness of outcome data, selective reporting, and other biases for each included study. In particular, we will assess other bias categories with particular emphasis on baseline imbalance between experimental and control groups, such as participant characteristics, including mean age, sex, or disease severity, because baseline imbalance in factors that are strongly related to outcome measures can cause bias in the estimation of the intervention effect in RCTs. We will categorize judgement relating to the risk of bias into 1 of 3 groups: “low risk,” “unclear risk,” or “high risk.” Each evaluation will be recorded in an Excel 2007 (Microsoft) spreadsheet and will be shared among researchers in Dropbox (Dropbox, Inc) folders. We will present the evaluated results in a full review using Review Manager version 5.3 software (Cochrane, London, UK).

We will evaluate the quality of evidence for each main outcome using the Grading of Recommendations Assessment, Development, and Evaluation approach.^[33]^ We will assess the risk of bias; inconsistency, indirectness, and imprecision of the results; and the probability of publication bias using a 4-part scale (“very low,” “low,” “moderate,” or “high”). We will present the results of the quality of evidence through a Summary of Findings table and the evaluation process will be shared among researchers using the online program GRADEpro (https://gradepro.org/).

### 2.7 Data synthesis and analysis

Data synthesis and analysis will be performed using Review Manager version 5.3 software (Cochrane) and will be shared among researchers in Dropbox (Dropbox, Inc) folders. In particular, for studies targeting PCS, we will analyze the efficacy of HM with separate comparisons. We will conduct descriptive analyses of the details of participants, interventions, and outcomes for all included studies. Meta-analysis will be performed if there are studies using the same types of intervention, comparison, and outcome measures. We will use risk ratio with 95% confidence intervals (CIs) for binary outcomes and mean difference or standardized mean difference with 95 CIs for continuous outcomes.

We will assess clinical heterogeneity by comparing the distribution of important participant factors (such as age, sex, disease severity, and specific types of TBI) and intervention factors (such as cointerventions and control interventions) between the included studies. In addition, we will assess statistical heterogeneity using both the chi-squared test and the I-squared statistic, a quantity that describes the approximate proportion of variation in point estimates due to heterogeneity rather than sampling error. We will consider I-squared values ≥50% and ≥75% that are indicative of substantial and high heterogeneities, respectively. In the meta-analyses, a random effects model will be used when the heterogeneity is significant (I-squared values ≥50%), while a fixed effects model will be used when the heterogeneity is not significant. Additionally, a fixed effects model will be used when the number of studies included in the meta-analysis is less than 5, in which case estimates of inter-study variance have poor accuracy.^[34,35]^

#### 2.7.1 Subgroup analysis

If the necessary data are available, we will conduct a subgroup analysis to explain the heterogeneity or to assess whether the treatment effects vary between subgroups according to the following criteria:

(1) different objectives of interventions, such as acute management or rehabilitation according to time frame following injury;
(2) severity of TBI; and
(3) different target symptoms of TBI, such as headache, dizziness, cognitive disorder, or mental disorder.

#### 2.7.2 Sensitivity analysis

To identify the robustness of the meta-analysis result, we will conduct sensitivity analyses by excluding

(1) studies with high risks of bias and
(2) outliers that are numerically distant from the rest of the data.

#### 2.7.3 Assessment of reporting biases

We will assess reporting biases, such as publication bias, using funnel plots if more than 10 studies are included in the meta-analysis. When a reporting bias is implied by asymmetry of the funnel plot, we will try to explain possible reasons.

### 2.8 Ethics and dissemination

Ethical approval is not required because this protocol is for a systematic review, not a clinical study. The results will be disseminated by the publication of a manuscript in a peer-reviewed journal or presentation at a relevant conference.

## 3 Discussion

Although TBI has been considered a major global cause of death and disability,^[2]^ effective treatment strategies for TBI are still in need of improvement. Several studies have suggested that HM, one of the CIM modalities, can be an effective alternative for managing TBI through multidimensional effects such as reducing brain water content, improving blood–brain barrier permeability, and reducing tumor necrosis factor-α/nitric oxide expression.^[20]^ In this regard, HM also has the potential to overcome the limitations of neurorestoration and neurorehabilitation that conventional management has.^[12]^ To date, some clinical evidence on the role of HM in the management of TBI has been documented; however, there is no systematic and critical review to evaluate the efficacy and safety of HM for TBI and/or PCS.

We believe that this systematic review will help clinicians establish optimal TBI and/or PCS management strategies. Furthermore, by identifying frequently used treatment strategies, such as the herb used and the duration of administration, this study will provide the basic research foundation for the development of optimal protocols for HM for TBI and/or PCS and the development of new drugs for these conditions in the pharmaceutical industry. In addition, as TBI is becoming a national issue due to the social and economic burden it causes, our review results will provide data that can be referred to during the formulation of national policies. Finally, the results of our review will contribute to expanding “health care choices” by providing TBI and/or PCS patients and their families with new management options that potentially complement conventional management strategies.

## Author contributions

The study was conceptualized by JL. The search strategy was developed by BL, JL, and CYK. The protocol was drafted by BL and CYK. JL, HK, HGJ, SHY, AS, JUS, YYC, and YY revised the manuscript. CYK submitted the manuscript for publication. All authors have read and approved the final manuscript.

**Conceptualization:** Jungtae Leem.

**Funding acquisition:** Jungtae Leem, Hee-Geun Jo.

**Methodology:** Boram Lee, Jungtae Leem, Chan-Young Kwon.

**Supervision:** Chan-Young Kwon.

**Writing – original draft:** Boram Lee, Chan-Young Kwon.

**Writing – review and editing:** Jungtae Leem, Hyunho Kim, Hee-Geun Jo, Sang-Hoon Yoon, Aesook Shin, Jae-Uk Sul, Ye-Yong Choi, Younghee Yun.

Jungtae Leem orcid: 0000-0003-3300-5556.

## Supplementary Material

Supplemental Digital Content
